# Laccase engineering by rational and evolutionary design

**DOI:** 10.1007/s00018-014-1824-8

**Published:** 2015-01-14

**Authors:** Isabel Pardo, Susana Camarero

**Affiliations:** Centro de Investigaciones Biológicas, CSIC, Ramiro de Maeztu 9, 28040 Madrid, Spain

**Keywords:** Laccase, Site-directed mutagenesis, Random mutagenesis, Directed evolution, Computational simulation, Protein engineering

## Abstract

Laccases are considered as green catalysts of great biotechnological potential. This has attracted a great interest in designing laccases a la carte with enhanced stabilities or activities tailored to specific conditions for different fields of application. Over 20 years, numerous efforts have been taken to engineer these multicopper oxidases and to understand their reaction mechanisms by site-directed mutagenesis, and more recently, using computational calculations and directed evolution tools. In this work, we review the most relevant contributions made in the field of laccase engineering, from the comprehensive study of their structure–function relationships to the tailoring of outstanding biocatalysts.

## Introduction

**Fig. 1:**
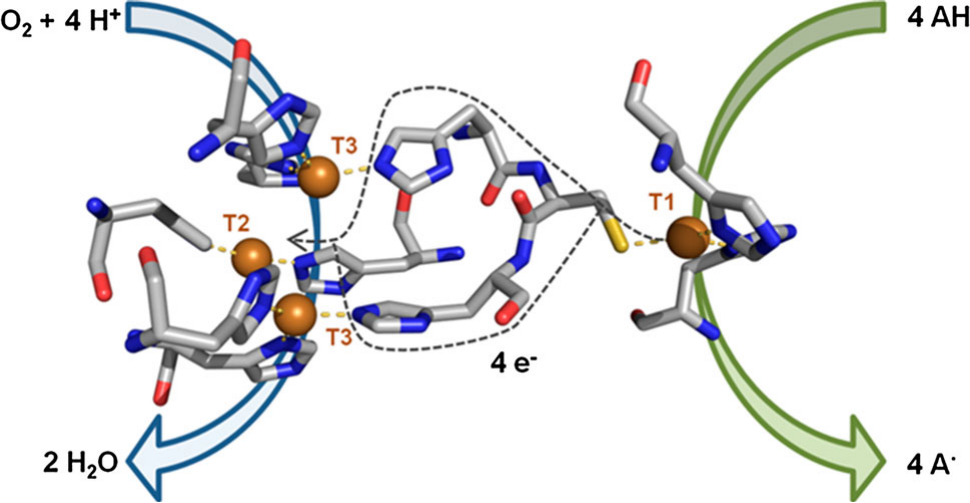
Catalytic site of *Pycnoporus cinnabarinus* laccase (PDB 2XYB) showing the Cu sites for oxidation of the reducing substrate (T1), and for reduction of O_2_ (T2/T3) and proposed electron transfer pathway between both sites. Catalytic coppers are shown as *spheres* and coordinating residues are shown as *sticks*

Laccases catalyze the one-electron oxidation of a set of aromatic compounds at the T1 Cu site, coupled to the four-electron reduction of oxygen to water at the trinuclear cluster (TNC) (Fig. 1). Even though they are widely distributed in nature, laccases produced by wood rotting and litter decomposing fungi are the most interesting from a biotechnological point of view due to their higher oxidative capabilities [1].

The crystal structures of a number of laccases and related multicopper oxidases (MCOs) have been solved in the last two decades [3]. Typically, laccases show a three cupredoxin-domain folding although small laccases with only two domains (SLAC) have been described in *Streptomyces* [2]. The combination of these data with site-directed mutagenesis and computational studies has provided valuable information regarding the main structure–function relationships in these enzymes. Yet, some aspects of the reactivity of laccases remain as subjects of debate, such as how the range of redox potentials or the diverse substrate affinities is tuned among laccases, thus hindering their rational design.

**Fig. 2:**
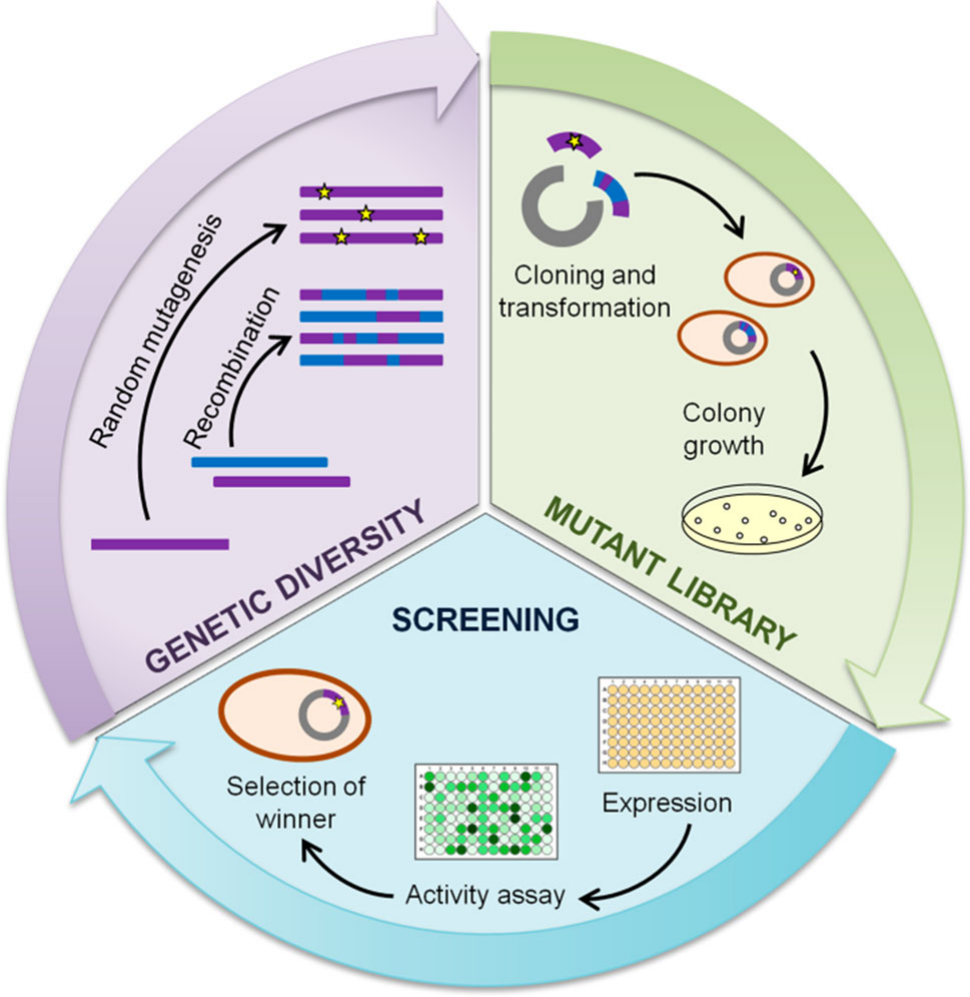
Schematic representation of a directed evolution cycle comprised by three main steps: generation of genetic diversity, expression of the mutant library and screening and selection of the best mutants

In recent years, the demonstrated potential of laccases in a range of applications has motivated the progress of laccase engineering efforts. Directed molecular evolution is an extremely powerful approach to tailor enzymes a la carte for particular purposes of application by mimicking in the lab the key processes of natural evolution. Genetic diversity is first generated by means of mutagenesis and/or recombination of a parent gene or a family of related genes. Once this variety is expressed in an adequate host, the mutant libraries are screened under a “selective pressure”. The selected mutants are subjected to new evolution rounds until the desired property is achieved (Fig. 2). Directed evolution does not require previous knowledge of the protein structure or reaction mechanism, but it requires significant screening effort for the analysis of thousands of clones. The availability of reliable screening methods to differentiate the best mutants from the rest is one of the main bottlenecks for evolutionary design. The preferred high-throughput screening (HTS) methods for the directed evolution of laccases have been, so far, based on colorimetric activity assays [4, 5].

Laccases are enzymes particularly suited for directed evolution. First, they are quite stable, which allows the accommodation of a fair number of mutations without obtaining an overly unstable protein. Second, they are catalytically versatile, so their activity can be enhanced towards a substrate that is not naturally well oxidized. Besides, the huge diversity of these enzymes allows one to choose the most suited scaffold for directed evolution. However, the difficult functional expression of recombinant laccases, particularly of fungal laccases, frequently makes their heterologous expression a target in itself for directed evolution prior to dealing with the improvement of activity/stability or specificity towards target compounds. Computational studies using protein structure prediction algorithms, molecular dynamics (MD) and hybrid quantum mechanics/molecular mechanics (QM/MM) calculations might aid the design of new laccase variants by reducing the experimental effort required to get the desired properties.

Here, we review the main inputs on rational and random design of laccases that have contributed to better understand the protein structure–function determinants and to improve the biotechnological applicability of these biocatalysts.

## From the understanding of the reaction mechanism

### Redox potential of T1 copper site, the role of the axial ligand

The T1 Cu center in bacterial laccases, as well as in the single blue copper proteins plastocyanin, azurin or rusticyanin, presents a distorted tetrahedral geometry constructed from a strong trigonal ligation (His-Cys-His) and a relatively weaker ligand in the axial position, generally a Met. In MCOs such as fungal laccases, ceruloplasmin or Fet3p, the Met ligand is replaced by a non-coordinating hydrophobic Leu or Phe residue, leading to a trigonal planar geometry [6].

The role of the axial ligand in tuning the redox potential of the T1 Cu centers has been extensively discussed due to the striking differences found among the different blue copper proteins. On one end, there is stellacyanin, a single blue copper protein with a redox potential (*E* ^0^) as low as +184 mV, which holds a Gln residue as axial ligand. On the other end, fungal laccases, ceruloplasmins and ferroxidases, all holding a Leu or Phe residue, have redox potentials roughly between +500 and +800 mV. However, the small blue copper protein rusticyanin, with a redox potential over +600 mV, shows a Met as axial ligand of the T1 Cu center. When the axial Met was replaced by Leu, the redox potential of rusticyanin raised 100 mV, while it decreased by a similar amount if Met was replaced by Gln [7].

The hydrophobicity of the axial ligand seems to correlate with the redox potential of the T1 Cu site in laccases [8], so that it might be considered as a rough indicator of their redox potential [3]. Laccases from plants (e.g., *Rhus vernicifera*) and bacteria (e.g., CotA laccase from *Bacillus subtilis* or SLAC), with a Met as axial ligand, show the lowest redox potentials (below +500 mV). Middle-potential laccases (up to +700 mV) mainly comprise ascomycete laccases and in general, with the exception of some basidiomycete laccases, have a Leu as non-coordinating axial ligand. Finally, high-redox potential laccases from basidiomycete fungi, with *E* ^0^ ≈ +790 mV, commonly have a Phe residue in this position.

First site-directed mutagenesis studies on fungal laccases showed the exchangeable contribution to T1 Cu *E* ^0^ of Phe and Leu as non-coordinating axial ligands. The replacement of the non-coordinating axial Leu ligand by Phe in *Rhizoctonia solani* (*E* ^0^ = +710 mV) and *Myceliophthora thermophila* laccases (*E* ^0^ = +470 mV) did not produce a significant increase in their redox potentials nor in the kinetics of the reaction [9]. Likewise, no significant alteration of *Trametes villosa* laccase properties was observed by changing the non-coordinating Phe axial ligand by Leu. By contrast, Phe replacement to Met resulted in 100 mV redox potential decrease, distorted EPR spectrum, and modified optimum pH and kinetic constants during oxidation of phenolic substrates [10]. These effects were attributed to a perturbation of the geometry of T1 site. Accordingly, the electric state of the T1 Cu center of the MCO CueO from *E. coli* became similar to those of fungal laccases by changing the Met axial ligand to Leu, obtaining also significant increase of the redox potential [11]. Substitution of the axial Met with Leu or Phe in *B. subtilis* CotA laccase increased the redox potential by 100 mV, attributed to the weakening in the T1 Cu coordination [8], but at the same time, a major drop of the enzyme activity was observed because the electron transfer between T1 Cu and the TNC became unfavorable [6]. Interestingly, the Phe mutant laccase underwent an intense drop of thermodynamic stability due to the loss of copper from the T1 site, indicating that copper depletion is a key event in the inactivation of the enzyme [8].

Significant perturbation of the electronic structure of *M. thermophila* laccase T1 Cu site was obtained by changing the non-coordinating axial Leu to His. The tetragonal distortion of the T1 site led to a σ overlap between the Cu and Cys(S) orbitals and to the green color of the His variant. The increased charge donation of the axial His coupled with the tetragonal distortion of the T1 site stabilized the oxidized state, hence lowering the redox potential (by 30 mV) and the reactivity of this site (tenfold decrease of *k*_cat_ respecting the blue wild-type laccase) [12]. Directed mutagenesis of the axial Met ligand to His in another MCO, namely bilirubin oxidase, caused the loss of copper and the lack of activity. When the Met ligand was changed to Gln, a coordinating residue not naturally found in MCOs [6], T1 Cu parameters resembled those of single-copper stellacyanin, accompanied by a remarkable drop in the enzymatic activity and a ~200 mV decrease in the redox potential [13, 14].

Simulation studies corroborated the crucial role of the axial ligand in defining the chemistry and redox potential of T1 site. QM calculations of six T1 Cu sites (cucumber stellacyanin, *Pseudomonas aeruginosa* azurin, poplar plastocyanin, *Coprinus cinereus* laccase, *Thiobacillus ferrooxidans* rusticyanin, and human ceruloplasmin) confirmed that the low redox potential of stellacyanin was mainly due to the Gln ligand at the axial position, whereas the presence of a non-coordinating hydrophobic residue contributes significantly to the increased redox potentials in *C. cinereus* laccase and human ceruloplasmin [15]. QM/MM and MD simulations data from *Trametes versicolor* laccase, CueO, CotA and SLAC also correlated at least in part the redox potentials with the hydrophobicity of the T1 Cu axial ligand [16].

The redox potential of T1 Cu centers is, however, tuned by other factors. One of them, first hypothesized by Piontek and co-workers [17], is the T1 Cu-His ligand distance. The elongation of the Cu-His(N_δ_) bond would account for a more electron-deficient copper and, consequently, for the observed higher redox potential in *T. versicolor* laccase as compared to *C. cinereus* laccase. Data from the crystal structure of *Rigidosporus lignosus* laccase [18] and MD simulations with CotA and SLAC [16] were mostly consistent with this hypothesis. However, although the protein fold could, in principle, modulate the T1 Cu redox potential by dictating the positions and orientations of the Cu ligands and adjusting the coordination bond strengths [15], overall, the relative changes in the Cu-ligand distances within the rigid His(N_δ_)-Cys(S)-His(N_δ_) environment can be assumed small, having a minimal effect on the T1 Cu redox potential for laccases. Conversely, the redox potential in MCOs is also influenced by the solvent accessibility, dipole orientation and H-bonding outside the T1 Cu coordinating sphere. T1 Cu redox potential would increase with N_backbone_(H)-Cys(S) H-bonding, whereas backbone dipoles would increase the redox potential and dipoles between side-chain and solvent decrease it [16].

### Substrate binding pocket

**Fig. 3:**
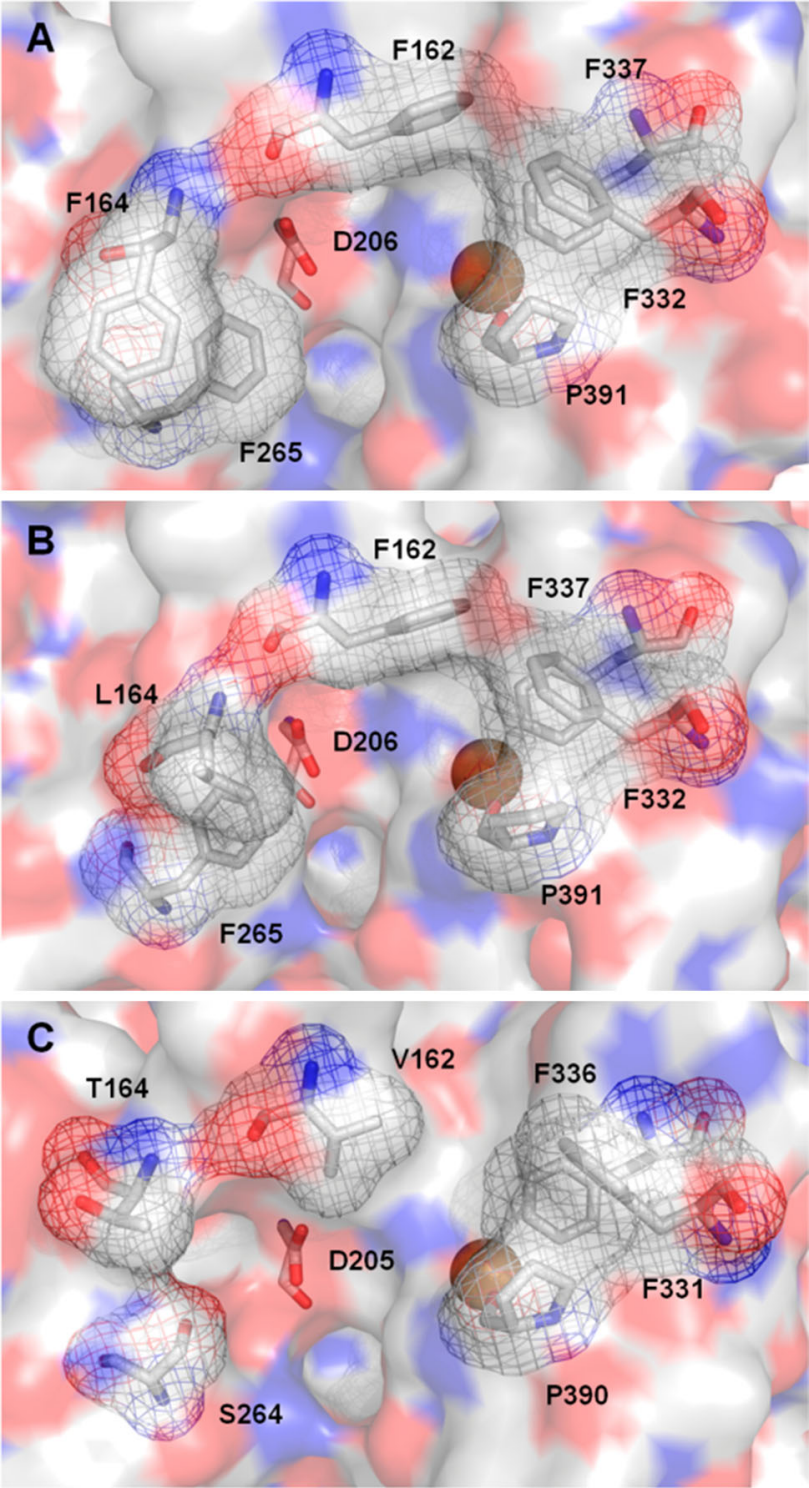
Close-ups of the substrate binding pockets of **a** *Pycnoporus cinnabarinus* (PDB 2XYB), **b** *Trametes versicolor* (PDB 1KYA) and **c** *Trametes trogii* (PDB 2HRG) laccases

One of the main issues regarding the oxidation activity of laccases is the interaction between the substrate and the enzyme. Laccases with high sequence identity and a striking structural similarity might show quite different residues delimiting the substrate binding pocket (Fig. 3), although a comparable accommodation of the substrate is largely preserved by a similar spatial conformation, hydrophobic nature and recognition properties of the binding pockets. Even so, the contribution of the residues of the substrate binding pocket to the oxidation capability of these generalist oxidases is clearly indicated by the variety of substrate binding sites and the different kinetic behaviors of laccases with similar redox potentials [19].

Xu and co-workers [9] first evidenced the dependence of substrate binding and electron transfer on the features of the enzyme pocket. The triple mutation of a tripeptide that is part of the substrate binding pocket in *R. solani* (*E* ^0^ = +710 mV) and *M. thermophila* (*E* ^0^ = +470 mV) laccases (LEA and VSG, respectively) caused a remarkable drop in *k*_cat_ and increase of *K*_m_ but no change in the redox potential of T1 Cu. These changes were attributed to electrostatic and steric hindrances to substrate docking introduced upon mutation.

The relative contribution of steric and redox features of putative substrates in determining their susceptibility to laccase oxidation was investigated by Tadesse and co-workers [20] in *T. villosa* (*E* ^0^ = +790 mV) and *M. thermophila* (*E* ^0^ = +470 mV) laccases. In spite of Δ*G* ^0^ between the substrate and T1 Cu site being the rate-determining reaction step, some of the substituted phenols and anilines investigated failed to be oxidized by the enzyme, even if they had a well-suited redox potential. A relationship between phenolic maximum dimension and substrate consumption was put forward. A steric threshold of 11–12 Å was reportedly dependent upon the distance between two Phe residues, Phe332 and Phe265, which mark the entrance to the active site. To overcome the protein steric restraints and improve its activity towards bulky phenols, four Phe residues of the substrate binding pocket of *T. versicolor* laccase (TvL) were subjected to site-directed mutagenesis [21]. Overall, the amino acid changes of the enzyme pocket mostly conditioned interactions needed for the correct orientation and binding of the substrate and the electron transfer reactivity. For instance, F265A mutation did not improve the oxidation of any of the bulky phenols tested due to the lack of proper hydrophobic interactions between the phenolic substrate and the replaced residue. In fact, the requirement of Phe in position 265 to bind and stabilize bulky hydrophobic aromatic compounds in the substrate binding site of TvL had been previously suggested [22]. The hydrophobic recognition of the substrate by the hydrophobic residues delimiting the enzyme pocket is supposed to be the first step of the mechanism of interaction between xylidine and laccase [23].

The H-bonding of the substrate with an Asp (or Glu) residue fully conserved in fungal laccases and located at the bottom of the binding pocket is also an important structural feature of the active site (Fig. 3) [17, 24–26]. The carboxylate group (Asp206 in TvL) is deprotonated at physiological pH (pKa 3.9). Hence, substrates bearing –OH and –NH_2_ groups are dragged inside by this negative charge and directed to the His ligand (His458 in TvL). While the electron is transferred to T1 Cu through the His ligand, the Asp residue assists the deprotonation of the substrate, thus providing a concerted electron/proton transfer mechanism for the oxidation of phenolic substrates [27]. The lower efficiency of TvL to oxidize phenolic compounds at pH 3 has been attributed to the poorer ability of the protonated Asp206 to drag the substrate inside the active site [20]. The role of the conserved carboxylic group in fungal laccases to assist the abstraction of the proton from the phenolic group of the substrate was demonstrated by directed mutagenesis studies in TvL [28] and in *Melanocarpus albomyces* laccase (MaL) [29]. A substantial decrease of TvL catalytic activity for 2,6-dimethoxyphenol (DMP), mainly due to remarkable increase of *K*_m_, was obtained by replacing the Asp for Asn or Ala residues. Besides, Asp206 stabilized the incipient radical cation of 2,5-xylidine resulting from the electron withdrawal by His458 [28]. Likewise, *k*_cat_ dropped 30-fold in MaL for DMP oxidation when Glu235 was changed for Thr [29].

Although the change of the acidic residue by non-acidic ones produced a shift of the optimum pH to more neutral values for the oxidation of DMP, no shift was obtained for the oxidation of ABTS [28, 29]. The distinct effect of the point mutations for the enzymatic oxidation of both substrates might be explained by dissimilar oxidation mechanisms, with (DMP) or without (ABTS) proton transfer [30]. Electrostatic interactions with the protonated Asp of the protein active site appear to be crucial for anchoring the negatively charged ABTS, thus explaining the higher activity of fungal laccases towards ABTS at pH 2–3 [9]. This assumption has been recently confirmed by the correlation between the net charge of the laccase binding pocket and the *K*_m_ values for ABTS in four *T. versicolor* laccase isoenzymes [31]. In the computational simulations, the best binding modes were obtained with the isoenzyme with the smallest measured *K*_m_ for ABTS at pH 2. ABTS was bound in a semi-extended conformation with one buried sulfonate group interacting with protonated Asp206 and the other sulfonate interacting with the Arg161. Residue Asp205 was also analyzed in POXA1b from *Pleurotus ostreatus* by site-directed mutagenesis. Its replacement by Arg not only significantly worsened the catalytic properties towards ABTS, DMP and syringaldazine (SGZ), but decreased protein stability [32].

### Intramolecular electron transfer and oxygen reduction

It is generally accepted that O_2_ is reduced in the TNC in two consecutive 2-electron steps. In the first one, the donation of two electrons from the T3 Cu ions of the fully reduced enzyme to O_2_ generates the peroxy intermediate. In the second step, the donation of two electrons from the T1 and T2 Cu ions to obtain water renders the fully oxidized native (catalytic) intermediate of the enzyme [33, 34]. A different mechanism has been reported for SLAC from *S. coelicolor*. A Tyr residue (Tyr108) placed at the vicinity of T2 site can provide one of the four electrons required for the reduction of O_2_, leading to the transient appearance of a Tyr radical, as demonstrated by the absence of radical in Y108A and Y108F mutants. Tyr acts as a kinetic buffer of redox equivalents to prevent the generation of harmful reactive oxygen species when the reducing substrate is limited [35].

The T1 site is coupled to the TNC through the highly conserved Cys-His bridge that provides a rapid intramolecular electron transfer pathway (Fig. 1). However, the role of neighboring residues to regulate the intramolecular electron transfer from T1 to the TNC or to assist the reductive cleavage of the O–O bond is not completely elucidated. Site-directed mutagenesis of the His ligands of T3 Cu in Fet3p raised the covalency of the T1-Cys(S) bond and reduced its redox potential. By contrast, the redox potential of the T1 Cu in *R. vernicifera* laccase would not be controlled by the redox states of the TNC due to a mechanism for regulating the intramolecular electron transfer from T1 to TNC [36].

The intramolecular electron transfer proceeds together with a proton transfer, required for the reduction of O_2_ to water, via ionizable groups placed within the access and exit channels to the TNC. Despite the differences existing among MCOs for oxygen binding [37], two acid residues strictly conserved in MCOs have a crucial role in O_2_ reduction, as demonstrated by site-directed mutagenesis in *B. subtilis* CotA laccase [38–40], *E. coli* CueO [41–43], and yeast Fet3p [44, 45]. A Glu residue forms an H-bond with one His ligand of T3 Cu [8, 44, 46, 47]. The absence of this Glu residue at the entrance channel to the TNC of CotA laccase (E498L or E498T mutants) caused over 99 % activity loss due to a remarkable drop of *k*_cat_, whereas the E498D mutant retained enzymatic activity and showed similar oxygen affinity. These data suggested the important role of Glu (and in a lesser extent of an Asp) in this position in providing protons for the reduction of oxygen and also in its binding [40]. X-ray crystal structure analyses of *E. coli* CueO mutants proved that the H-bond network built by the Glu residue and water molecules acts as the pathway of proton relay from solvent waters to the TNC for oxygen reduction. The total collapse of the H-bond network in E506I mutant accounted for the complete loss of activity [42].

An Asp residue, which is also part of the H-bond network around the TNC in close proximity to T2 Cu, is a key residue as a proton donor to assist the reductive cleavage of the O–O bond, providing the rapid conversion of O_2_ to water [14, 39, 41]. First mutagenesis studies carried out on this residue in Fet3p (Asp94) demonstrated its role in the decay of the peroxide intermediate [45]. D94E mutation did not affect the initial reaction with O_2_ but markedly diminished the decay of the peroxide intermediate. The D94A mutation caused larger structural changes that made the TNC unreactive towards O_2_, demonstrating also a structural role for Asp94. Saturated mutagenesis of this residue (Asp116) in CotA laccase corroborated its importance. The absence of a carboxylate group impaired the decay of the peroxide intermediate and strong depletion of the catalytic activity, downshift of optimal pH and perturbation of the properties of T1 Cu were observed in all mutants [39]. However, neither the structure of TNC nor the oxygen affinity was affected upon mutation, by contrast to what was reported for *Myrothecium verrucaria* bilirubin oxidase, yeast Fet3p or *E. coli* CueO [14, 41, 44]. The negative charge of Asp116 (which remains deprotonated in the entire pH range) would supply protons to the –OH groups bound to T2 Cu and control the protonation of Glu498, the sole protonable residue in the vicinity of TNC [39].

## To the engineering of improved biocatalysts

### Enhancement of heterologous expression

Due to their fast growth rate, easy genetic manipulation and availability of numerous molecular biology tools, *E. coli* and *Saccharomyces cerevisiae* are the most commonly used expression hosts in protein engineering. However, very frequently, expression is hampered by differences between the organism of origin and the heterologous host, such as codon usage, chaperones to assist protein folding, and post-translational modifications (glycosylation, disulfide bonds, secretion signals, etc.). Heterologous expression is particularly troublesome in the case of fungal laccases, so that the increase of expression yields has constituted, in itself, a target for directed evolution.

Soluble expression in *E. coli* of the endospore laccase from *Bacillus licheniformis*, similar to *B. subtilis* CotA laccase, was enhanced 11-fold by a combination of random and site-directed mutagenesis [48]. One of the selected mutations (D500G) was found adjacent to the axial Met of T1 Cu, being responsible for an eightfold increase of soluble expression. An Asp residue in this position has only been found in laccases from *Bacillus* genus, whereas other bacterial and fungal laccases present a Gly. This mutation was later introduced in laccase from *Bacillus* sp. HR03, also giving rise to a threefold increased expression in *E. coli* [49].

In the case of fungal laccases, the preferred expression host for directed evolution has traditionally been *S. cerevisiae*. Heterologous expression of *M. thermophila* laccase (MtL) in this yeast was improved eightfold after ten rounds of error-prone PCR and in vivo shuffling [50]. Of all the mutations selected in the final evolved mutant, three mutations were located in different processing sites: two in the native signal propeptide and one in the C-terminus. In particular, the one at the C-terminus was the single mutation responsible for the highest total activity increase. It introduced a new KEX2 protease cleavage site, probably allowing C-tail trimming in the yeast host. C-tail processing has been described to be essential for activity in several ascomycete laccases [51, 52].

For the directed evolution of the high-redox potential laccases (HRPLs) from the basidiomycetes PM1 (PM1L) and *Pycnoporus cinnabarinus* (PcL), their cDNAs were fused to *S. cerevisiae*’s alpha mating factor prepro-leader sequence [53, 54]. By performing random mutagenesis over the whole fusion gene, expression and activity could be enhanced simultaneously. In combination with other mutations in the mature laccase sequence that could also favor expression in yeast (including synonymous mutations that increased codon usage), mutant signal peptides provided expression yields of up to 8 mg/L. In particular, the evolved alpha-factor prepro-leader obtained during the directed evolution of PcL alone was responsible for a 40-fold increase in expression.

The evolved prepro-leaders showed similar mutations in the hydrophobic core of the preleader (A[α9]D in PcL and V[α10]D in PM1L evolved variants), which is involved in the translocation of the nascent polypeptide to the ER. These mutations could be exchanged between both laccases, giving rise to similar increases in extracellular activity [54]. Another similarity was found in the spacer sequence between the alpha-factor prepro-leader and the mature laccases, which presents the motif EAEA for cleavage by STE13. In both PcL and PM1L evolved variants, the EAEA motif was disrupted by mutations E[α86]G and A[α86]T, respectively. N-terminal sequencing of PM1L evolved variant confirmed the extension of the N-terminus by six amino acids. This misprocessing by STE13 resulted beneficial as demonstrated by the 40 % decrease on laccase secretion when a truncated variant without these extra amino acids was produced [55].

### Improving the catalytic activity and substrate specificity

**Fig. 4:**
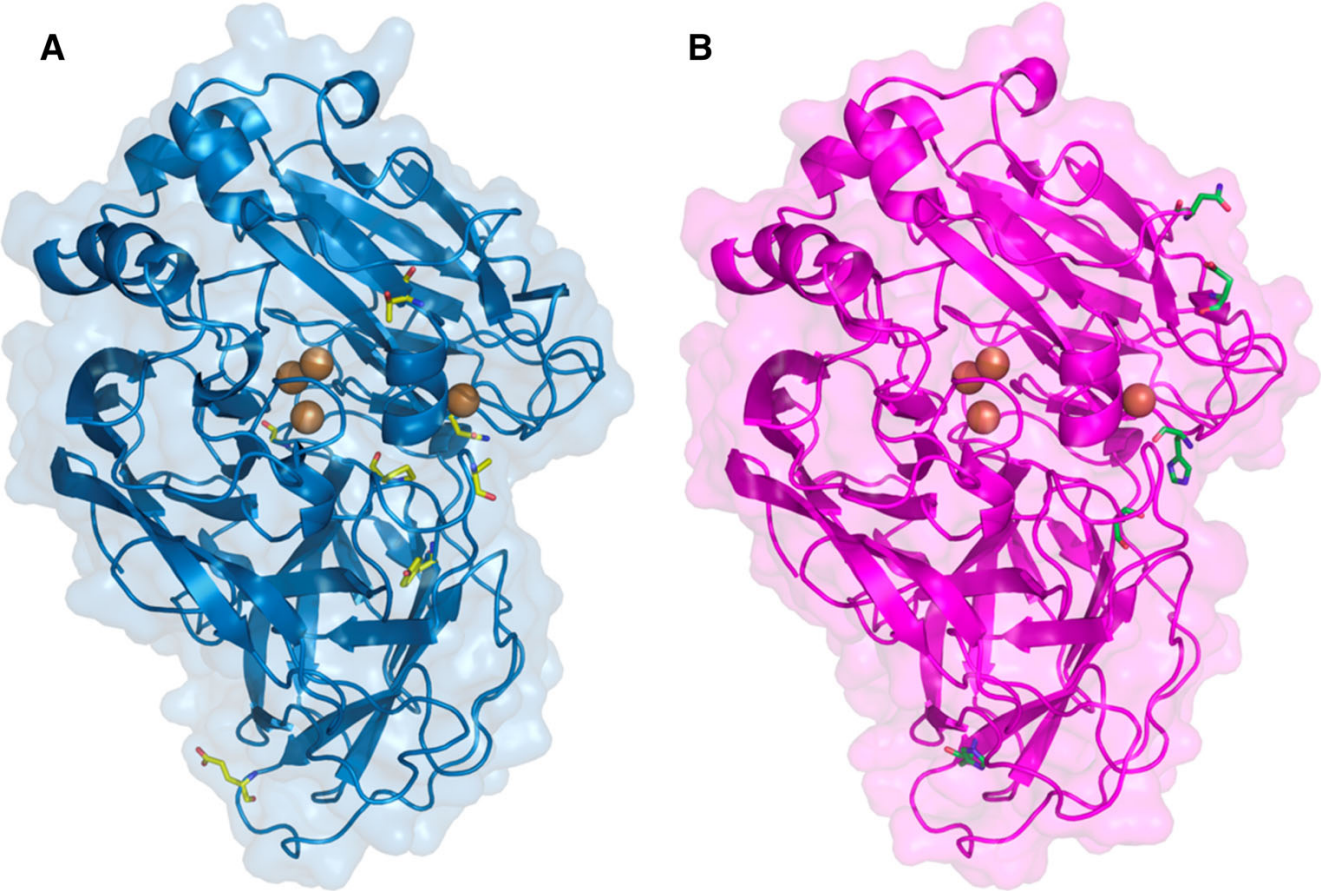
Protein models of the evolved laccase variants from **a** basidiomycete PM1 and **b** *Pycnoporus cinnabarinus*. Models are based on 2HRG and 2XYB PDB structures, respectively. Mutations accumulated through evolution are shown as *sticks*

The advances made in the knowledge of laccase structure–function relationships and the availability of quite a number of laccase crystal structures have facilitated the rational design of laccases for specific targets. It can be assumed that substitutions on amino acids located in the substrate binding pocket or in the vicinity of the catalytic coppers will affect activity. However, beneficial mutations located far from these sites have also been found to affect the overall enzyme activity in directed evolution studies (Fig. 4), highlighting the power of directed evolution to reveal new targets for protein engineering.

The substrate binding pocket of SLAC from *S. coelicolor* has been re-designed by site-directed mutagenesis to improve its activity towards compounds of interest as redox mediators. The replacement of the two Met of the pocket by small residues (Ala or Gly) remarkably increased the catalytic efficiency with DMP and the decolorization of indigo carmine mediated by methyl syringate or TEMPO [56]. The residues of the substrate binding pocket of CotA laccase were randomly modified by saturated mutagenesis to increase the specificity of the enzyme for ABTS over SGZ. The final mutant laccase (G417L and L386W) was 132 times more specific than the wild-type enzyme. Both mutated residues sandwiched ABTS, thus providing favorable interactions that would enhance the binding of the substrate [57]. In our group, we are re-designing the substrate binding pocket of a chimeric laccase obtained by DNA shuffling of two fungal HRPLs [58] using combinatorial saturation mutagenesis. Mutant libraries are being screened for enhanced activity towards phenolic compounds derived from lignin [5] which might be used as natural mediators of laccases in the lignocellulose bio-refineries [59].

The C-terminal tail plays also a critical role on laccase activity. Several site-directed mutagenesis studies have shown that ascomycete laccases typically present a C-terminal extension that acts as a plug that blocks access to the water channel. The crystal structure of *M. albomyces* laccase (MaL) revealed the penetration of the final four residues into the entrance channel to the TNC, forming an H–bond with one of the His residues that coordinate T3 Cu. The deletion of these four residues resulted in an almost inactive laccase, and substitution of the final Leu559 for Ala caused a threefold decrease in specific activity [51]. A C-terminal protrusion that blocks access to the TNC channel has also been described in two laccases from *P. ostreatus* (POXA1b) and *Pleurotus eryngii* (Ery4), which share 95 % sequence identity and present a 16-residue Ct-extension that is not found in other basidiomycete laccases. Curiously, while POXA1b is active when expressed heterologously in yeast, Ery4 is secreted in an inactive form (probably due to incorrect C-terminal processing). Truncated variants at the C-terminal tail were obtained for both laccases. In the case of POXA1b, truncation of 4 or 16 residues increased activity towards the phenolic substrates DMP and SGZ, but not towards ABTS [32]. On the other hand, deletions ranging from 2 to 18 residues in the C-terminus of Ery4 resulted in active variants, being the 5-less mutant the most catalytically efficient. Furthermore, Lys532 (second to last residue) was crucial for the regulation of enzymatic activity. As in MaL, this residue seems to form a network of H-bonds with residues near the TNC, probably immobilizing the C-terminus inside the water channel and obstructing it. Mutation of Lys532 for Ala or Glu restored laccase activity [60].

When the thermostable laccase from *M. thermophila* (MtL) was expressed in yeast, *k*_cat_ was reduced tenfold for ABTS and 25-fold for SGZ. After ten rounds of directed evolution, wild-type values were recovered for SGZ and doubled for ABTS and, due to the selective pressure introduced during library screening (pH 6), optimum pH for ABTS oxidation was shifted from 3 to 4. Surprisingly, of the ten mutations accumulated in the mature evolved laccase, only three of them were located near the catalytic copper ions (Y403H, A108V) or the enzyme pocket (N454K), while the rest were located in loops or on the protein surface. This first evolved variant has later been used as parent type in a number of directed evolution works for different purposes [61, 62]. In a semi-rational study aimed to enhance catalytic activity and to better understand the role of the C-terminal end in MtL activity, residues from several conserved regions were targeted for combinatorial saturation mutagenesis [63]. The only non-synonymous mutation selected after screening 180,000 clones was S510G from the VSG tripeptide of the substrate binding pocket [9], which resulted in higher *k*_cat_ values for ABTS and DMP and twofold increase in *K*_m_ for O_2_. This mutation causes the disruption of the interaction with the C-terminal plug, thus widening the access of the O_2_ channel to the TNC. In other evolution studies, two more mutations related to enhanced activity were selected: mutation N552H, located near the C-terminal plug and causing twofold increase in *K*_m_ for O_2_; and mutation L429V, found buried in the substrate binding pocket [64].

For the directed evolution of POXA1b laccase, three rounds of random mutagenesis were performed together with a round of site-directed mutagenesis [65–67]. The final evolved mutant, with five non-synonymous mutations, increased 3.5-fold its activity towards ABTS and twofold towards DMP, and presented higher affinity towards DMP. MD simulations were used to evaluate the effect of some of the mutations selected through the evolution pathway. Mutation L112F, selected in the first generation, was located at the entrance of the water channel that leads to the TNC, and Phe residue could act as a lid that trapped water molecules inside the channel favoring catalysis [65]. Mutation P494T, located at the C-terminal extension of POXA1b, seemed to increase mobility of the loops that form the substrate binding pocket, making the T1 Cu more solvent accessible. Lastly, mutation V148L appeared to affect catalysis by establishing stronger hydrophobic interactions with Tyr208, located in the loop where Asp205 (key to substrate binding) is found.

Random and semi-rational approaches were used in the parallel directed evolution pathways of laccases from basidiomycetes PM1 (PM1L) and *P. cinnabarinus* (PcL) [53, 54]. Due to the extremely low expression levels of these laccases in yeast, these studies aimed to increase both secretion and catalytic activity, addressing total activity increases (TAI) for mutant selection. After eight and six generations, TAIs of 34,000- and 8,000-fold were achieved for the evolved variants of PM1L and PcL, respectively. In the case of evolved PM1L, catalytic constants were similar to those of the wild-type enzyme from the fungus [55]. Comparison with the best mutant from the second generation revealed a 13-fold *k*_cat_ improvement for ABTS and ninefold for DMP, suggesting that laccase activity was initially hampered due to the heterologous expression in yeast (as it had been described for MtL [50]). The final evolved variant of PM1L presented seven non-synonymous mutations in the mature laccase sequence, three of which were located near the catalytic center (Fig. 4a). Val162, which is one of the residues that delimits the substrate binding pocket, was substituted for the also hydrophobic but smaller amino acid Ala, probably favoring binding of bulky substrates such as ABTS. Mutations S426N and A461T, found in the second coordination sphere of T1 Cu, established a new network of H-bonds that could change the geometry at the T1 site, affecting the catalytic activity.

As for the evolved PcL, an important broadening of the optimal pH towards higher values was observed as a consequence of the selective pressure applied during the screening of the mutant libraries, as described for other laccases [50, 65]. By contrast to PM1L, the activity of the native PcL was not affected by the expression in yeast. Even so, *k*_cat_ values for ABTS, sinapic acid and DMP were considerably enhanced through evolution, from 10- to 20-fold, with regard to wild-type and native recombinant PcL [53]. The evolved laccase accumulated five amino acid substitutions, three of them considered to be directly related to enhanced activity (Fig. 4b). Mutations N208S and N331D, located at two of the loops that define the substrate binding pocket, could affect substrate recognition. Mutation P394H, selected in the first generation with a TAI of fivefold, is adjacent to His395 that coordinates T1 Cu. This mutation was introduced by site-directed mutagenesis during the engineering of PM1L, causing an important increase in activity (although it was finally discarded due to a somehow negative effect on thermostability). Site-directed mutagenesis studies currently being carried out in combination with computational simulations corroborate the crucial role of P394H mutation in the activity enhancement of the final evolved PcL (unpublished data).

### Improvement of laccase stability

For the industrial application of laccases, robust enzymes that are stable and active under harsh operational conditions are needed. Though some studies aimed to increase stability of laccases by site-directed mutagenesis have been reported, to date probably the most significant advances have been achieved by directed molecular evolution, as the structural determinant for protein stability may not be so straightforwardly deduced. By developing appropriate HTS methods, increased stability towards organic solvents, high temperature, or extreme pH can be obtained after several rounds of directed evolution.

The thermostable POXA1b laccase exhibits a notable stability at alkaline pH due to the extended C-terminal tail (in particular, the last four residues). Truncated mutants drastically lost the stability at pH 10, while they showed an increased stability at pH 5 [32]. The same effect was observed in truncated variants of Ery4 laccase from *P. eryngii*, for which progressive deletion of residues from the C-terminal tail led to an increased stability at acidic pH. Thermostability was also affected, but there was no direct correlation between the number of residues that were deleted and increased tolerance to high temperature [68]. During directed evolution of POXA1b, mutants with increased stability were obtained. Mutation L112F, selected in the first generation, caused an important decrease in laccase stability. MD simulations showed that the substitution of Leu residue for a bulkier Phe caused an increased flexibility of the entire subdomain. This effect was, however, reverted in the following generation thanks to mutation P494T, which seemed to recover rigidity in this region [65]. When these mutations were combined with those of another stable mutant (K37Q K51N), the stability of both parents towards temperature and pH 10 was inherited and even increased [66].

It is widely accepted that there is a trade-off between enzymatic activity and stability, and very frequently in directed evolution studies mutations that enhance catalysis are destabilizing for the protein. For this reason, it is interesting to introduce stability assays in conjunction with the standard activity assays for the screening of mutant libraries, to enhance stability or to detect destabilizing mutations. During the evolution of basidiomycete PM1 laccase, the use of a thermostability assay [69] enabled the authors to find the mutation responsible for the loss in enzyme stability. Mutation F454S, adjacent to His455 that coordinates T1 Cu, improved total activity almost fivefold but at the cost of a decrease of 5 °C in T_50_ (the temperature at which the protein retains 50 % of its initial activity after a 10-min incubation) versus parent types [54]. Although successive evolution rounds further increased activity, they did not recover parent T_50_, so this mutation was finally reverted. This way, the final evolved mutant maintained the same T_50_ as the wild-type laccase (around 73 °C).

Zumárraga et al. described the directed evolution of MtL for increased activity and stability in the presence of organic solvents. After five generations of directed evolution, in which they used a HTS assay based on the oxidation of ABTS with increasing concentrations of ethanol and acetonitrile [70], they achieved a laccase mutant active and stable in high concentrations (up to 50 %) of co-solvents of different chemical nature [62]. Two mutations in the mature protein, E182K and S280N, were found to establish new stabilizing interactions with surrounding residues.

### Chimeric enzymes

Recombination of related genes allows the exploration of a large sequence space without affecting the structure–function of a protein, leading to new variants with combined features or unexpected properties by accumulation of neutral mutations that increase the promiscuous functions or robustness of the enzyme [71]. Random recombination of genes by DNA family shuffling or rationally guided recombination after careful inspection of protein structures have been assayed on laccases.

Two chimeric laccases were obtained by exchanging the N-terminal and C-terminal halves of Lcc1 and Lcc4 isoenzymes from *Lentinula edodes*, in such a way that the resulting hybrids presented two Cu-binding motives from each parent [72]. Lcc1 is secreted in the culture media and presents high activity towards ABTS, while Lcc4 is accumulated in fruiting bodies and is more active towards phenolic compounds. Of the two chimeras, only Lcc4/1 was actively secreted by transformed tobacco cells. This new laccase presented low *K*_m_ values for phenolic compounds, similar to those of Lcc4 or even better. However, turnover rates were severely impaired (approximately tenfold respecting Lcc4), although this could be due to decreased reaction rates caused by heterologous expression. Its pH profile was intermediate to those of the parent laccases, with a similar but less pronounced acidic profile than that of Lcc4. Concerning temperature, all three laccases presented maximum activity at 50 °C. However, while Lcc4 had around 50 % relative activity at 60 °C, Lcc1 and Lcc4/1 retained 80 % activity.

Similarly, Bleve et al. described the exchange of N-terminal and C-terminal residues between Ery3 and Ery4 laccases from *P. eryngii* [73]. As described above, Ery4 presents sixteen extra residues at the C-terminus and is inactive when expressed in yeast. Ery3, however, does not present this extension and is functionally produced by yeast. The authors constructed three chimeric laccases based on Ery4 scaffold: Ery4 with N-terminus signal peptide from Ery3 (4N3), Ery4 with C-terminus from Ery3 (4C3), and Ery4 with both N- and C-termini from Ery3 (4NC3). All constructions produced active laccases, even 4N3 variant that maintained the C-terminal extension from Ery4. This highlights the important role of the N-terminus in determining active protein folding. Both chimeras with exchanged C-terminus doubled *k*_cat_ values of Ery3 for ABTS and retained activity at pH 8. 4NC3 was also the most efficient oxidizing phenolic compounds.

**Fig. 5:**
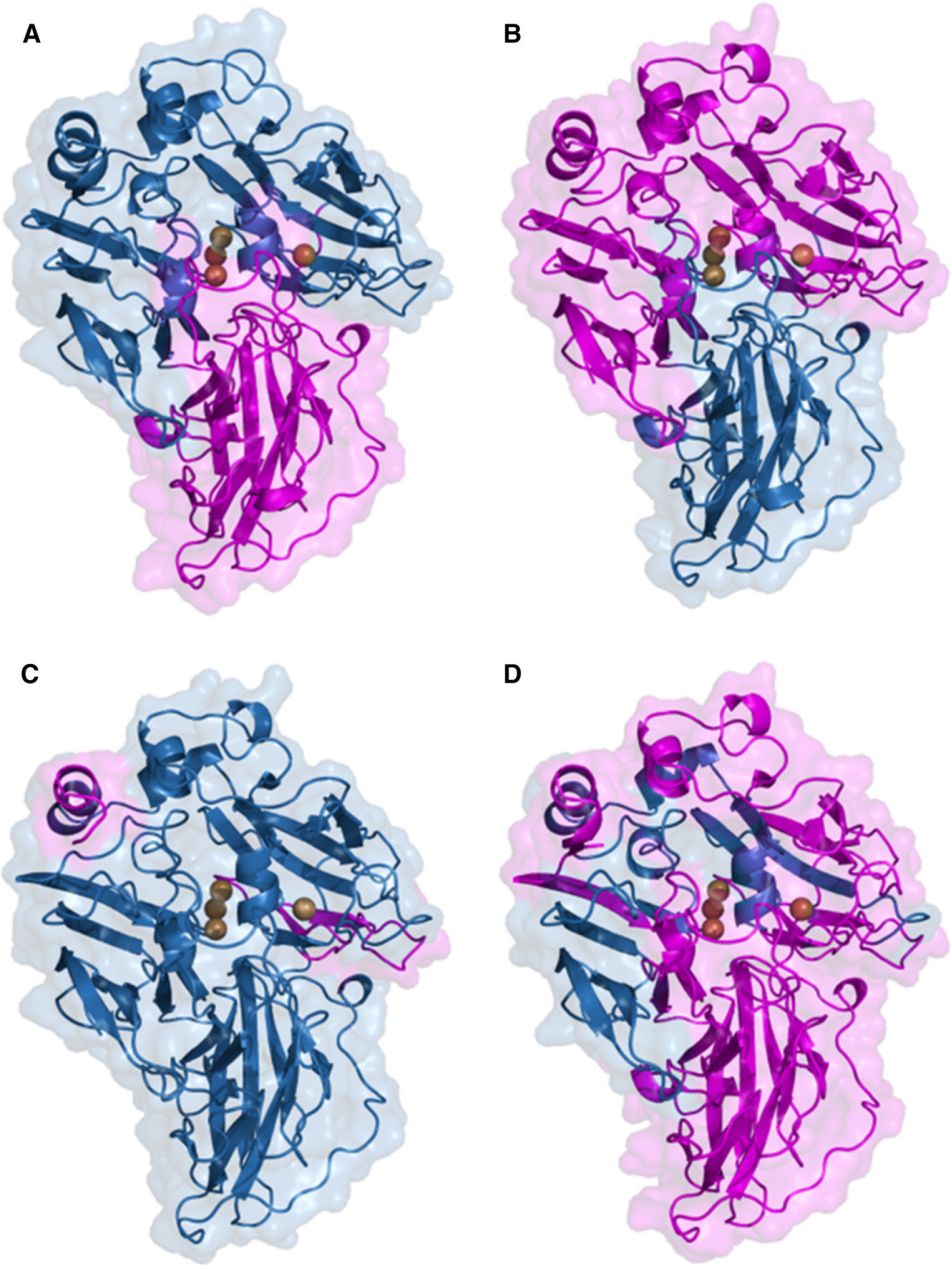
Protein models of chimeric laccases obtained by domain swapping (**a**, **b**) and random DNA shuffling (**c**, **d**) of PM1 (*blue*) and *Pycnoporus cinnabarinus* (*magenta*) evolved laccases, showing the fragments inherited from each parent. Models are based on 2HRG or 2XYB PDB structures

The evolved laccase variants of *P. cinnabarinus* (PcL) and basidiomycete PM1 (PM1L) have also been used to create hybrid laccases in a structure-guided manner by exchanging their D2 domains [74] (Fig. 5a, b). In this case, *K*_m_ values and pH profiles of the swapped mutants were intermediate to those of the parent laccases. Unexpectedly, the mutant containing D2 from evolved PcL showed an important increase of over 7 °C in its T_50_. Even though D2 from evolved PcL introduced a new glycosylation site in the swapped laccase, the increase in stability was caused only by the new interactions between amino acids, as removal of the N-glycosylation site by site-directed mutagenesis had no negative effect on T_50_.

DNA recombination can be easily achieved by taking advantage of the high homologous recombination frequency and efficient gap repair machinery of *S. cerevisiae*. Chimeric laccases from *Trametes* C30 *sp*. were obtained by this procedure by allowing the sequences for isoenzymes lac1, lac2 and lac5 to recombine individually with lac3, which was actively expressed in highest levels in yeast [75]. Due to the recombination strategy used, the three chimeras studied (namely LAC131, LAC232 and LAC535) presented a central region from LAC3 (around 250 residues on average) and the N- and C-terminal regions from the different laccases used as “acceptors” (the entire D1 and the final part of D3). All of them presented similar catalytic values to LAC3 at its optimum pH of 5.7, but LAC131 and LAC232 notably enhanced activity at basic pH values.

A combination of in vitro and in vivo DNA shuffling was used for random recombination of evolved PcL and PM1L (76 % sequence identity), and mutants with improved activity were selected in a dual HTS [58]. In this case, the number of crossover events in the mature protein sequence ranged from one to five, introducing 3-31 amino acid substitutions per clone respecting the most similar parent. All recombination events took place at D1 and D3, and many of them close to the conserved copper binding motifs (Fig. 5c, d). This was probably due to the lower sequence identity between the two laccases in D2 respecting D1 and D3 (67 versus 81 and 79 %, respectively). Chimeric laccases presented varied pH profiles for ABTS and DMP oxidation, shifted towards more acidic or more neutral pH values, and some of them even increased T_50_ of both parent laccases. Better affinities for DMP oxidation at pH 5 were also observed in some selected chimeras. Interestingly, these differences could be found in hybrid laccases that only exchanged a few final residues of the C-terminus, which again highlights its importance in modulating activity and stability.

### Laccase engineering beyond the natural limits

By presenting the enzymes to a selective pressure to which they had not been confronted in the course of their natural evolution, directed molecular evolution allows to obtain biocatalysts with properties that are not found in nature. Laccases typically perform their function in an acidic environment, so their optimum activity is generally found below neutral pH values. It is believed that the excess of OH^−^ ions inhibits electron transfer from T1 Cu to the TNC. However, some of the most important industrial applications of laccases require enzymes that are active at pH ≥ 7, such as paper pulp bleaching and decolorization of industrial dyes.

In several directed evolution studies, the pH activity profiles of the wild-type laccases were shifted in the evolved variants due to the selective pressure applied during mutant library screening [50, 53, 64]. Taking advantage of this fact, MtL mutant [64] was further evolved to enhance its activity at basic pH, while retaining its activity at pH 5, by performing a dual screening [61]. The final evolved mutant, obtained after 5 generations, retained 90 % of maximum ABTS oxidation rate at pH 4–6 and presented 50 % relative activity at pH 7, a value at which the parent type showed less than 10 %. In the case of DMP, optimum pH was shifted from 4 to 6, presenting around 80 % relative activity at pH 7. Turnover rates were increased 31-fold for ABTS and ninefold for DMP at pH 5. Of the mutations accumulated in the final variant, N109S, located near the TNC, seemed to be responsible for these improvements. This mutation disrupts H-bonds with several surrounding residues that include His140 (ligand of one of the T3 Cu) and G558 (part of the C-terminal plug which regulates O_2_ entry to the TNC).

The increasing interest in using laccases (in particular HRPLs) in nanodevices and biosensors resides in their uncommon capability for accepting electrons directly from cathodes, which makes them suitable for the development of biofuel cells required for implantable nanobiodevices. The main drawback is that HRPLs are generally inactive at physiological pH and are inhibited by chloride concentrations lower than those found in human blood (150 mM) by a similar mechanism to inhibition by OH^−^. Mate et al. obtained a blood-tolerant laccase by directed evolution [76], combining random, site-directed and saturation mutagenesis of the PM1L evolved variant [54]. They developed a “blood-buffer” for screening mutant libraries that simulated the chemical composition of human blood, gradually increasing buffer pH from 6.5 to 7.4 throughout four generations. The final evolved variant was active in human plasma and blood. Its pH activity profile notably shifted from 4 to 5–6 for DMP oxidation and was broadened for ABTS. At pH 7, it retained 50 and 20 % relative activity towards DMP and ABTS, respectively, while activity of the parent type was null. However, turnover rates decreased fourfold and threefold for these substrates at their respective optimum pH. Halide tolerance was also enhanced, raising the I_50_ for Cl^−^ (concentration at which activity is 50 % of the initial value) from 176 to 1,025 mM when ABTS was used as substrate at pH 4. At pH 7.4, no negative effect for ABTS oxidation was observed in the range of 100–800 mM NaCl. Both mutations selected in the final evolved mutant, F396I and F454E, were located near the T1 site, in the second coordination sphere. It was hypothesized that these mutations affect electron transfer from the substrate to the T1 (the rate limiting step), compensating the inhibition caused by OH^−^ and halides at the TNC.

## Concluding remarks

In spite of the huge biotechnological potential of laccases and the existence of some commercial preparations, the implementation of laccases at industrial scale is a pending issue. Today, the use of novel computational methods, and cutting-edge directed evolution techniques, supported by the know-how generated during the last two decades on laccase structure–function relationships, can change this scenario. In this endeavor, rational and evolutionary design will guide the engineering of recombinant biocatalysts better suited for target biotransformations under defined operational conditions. Computational simulations and directed evolution will interact to reveal targets for protein engineering to be explored by site-directed mutagenesis (or semi-rational approaches) or for designing smart libraries to reduce the screening efforts in evolutionary strategies.
